# Capillary-induced Homogenization of Matrix in Paper: A Powerful Approach for the Quantification of Active Pharmaceutical Ingredients Using Mass Spectrometry Imaging

**DOI:** 10.1038/srep29970

**Published:** 2016-07-21

**Authors:** Maico de Menezes, Diogo Noin de Oliveira, Rodrigo Ramos Catharino

**Affiliations:** 1 INNOVARE Biomarkers Laboratory, School of Pharmaceutical Sciences, University of Campinas, Campinas, 13083-877 SP, Brazil

## Abstract

Herein we present a novel approach for the quantification of active pharmaceutical ingredients (APIs) using mass spectrometry imaging. This strategy uses a filter paper previously “eluted” with a MALDI matrix solution as a support for analyte application. Samples are submitted to mass spectrometry imaging (MSI) and quantification through characteristic fingerprints is ultimately performed. Results for the content of rosuvastatin from a known formulation are comparable to those obtained with a validated HPLC method.

Quantitative analysis of Active Pharmaceutical Ingredients (APIs) plays a major role on the routine of pharmaceutical companies, for reasons that range from quality control^1^,^2^ and pharmaceutical development^3^ to compliance with regulatory demands^4^. Chromatographic approaches, such as HPLC and GC, are widely employed in these environments, and are aimed at controlling both APIs^5^, residual solvents^6^ and other related substances/impurities^7^ in tablets, capsules and a wide array of other dosage forms^8^. The main issue with the use of chromatography is mostly derived from the residues of solvents that are generated from the analyses; growing trends in green chemistry now lean toward the minimization of waste arising from routine analyses^9^.

Mass spectrometry (MS) has been increasingly employed as a powerful analytical tool that can be successfully implemented in pharmaceutical analyses^10^ for its great specificity in providing reliable results regarding structure information of target molecules in complex mixtures. Within the scope of MS, several important advances were developed in the last couple of decades, noticeably the use of soft ionization techniques such as MALDI coupled with mass spectrometry imaging (MSI) features^11^, to obtain valuable information on the spatial distribution of active ingredients directly from the tablet^12^. Other studies broadened this view, by showing that the quantification of degradation products of APIs is also possible directly in the tablet itself, with remarkable results, even when compared to standard methodologies such as HPLC^13^.

Analytical approaches based on MS that deal with complex samples usually rely on chromatography for cleaning and retaining undesired compounds, preventing them from entering the instrument. In this sense, sample-eluted paper has already been used as an ingenious and suitable support for samples submitted to MS analyses, with ionization methods similar to ESI – the paper spray approach^14^,^15^, with applications in biological samples^16^ and quantitative analyses^17^, rendering a successful technique that has since been developed to provide even better results. Following a similar trend, this contribution presents a novel approach for the quantification of active pharmaceutical ingredients (APIs) using mass spectrometry imaging. This strategy uses a filter paper previously “eluted” with a MALDI matrix solution that will serve as a “readily-ionizable”, homogenous support for further analyte application and analysis. The paper strips are submitted to MSI, followed by quantification through chemical images of the selective drug signal is ultimately performed, following the principles described in earlier contributions^18^,^19^.

## Experimental Section

### Rosuvastatin standard stock solution

A concentrated rosuvastatin standard stock solution was employed for achieving all subsequent dilutions used in the calibration curve, made up with 98.5 mg of rosuvastatin (96.1% of purity) in 20 mL of methanol:acetonitrile (1:1), to achieve a final concentration of 4.733 mg.mL^−1^.

### Sample preparation

**Figure 1:**
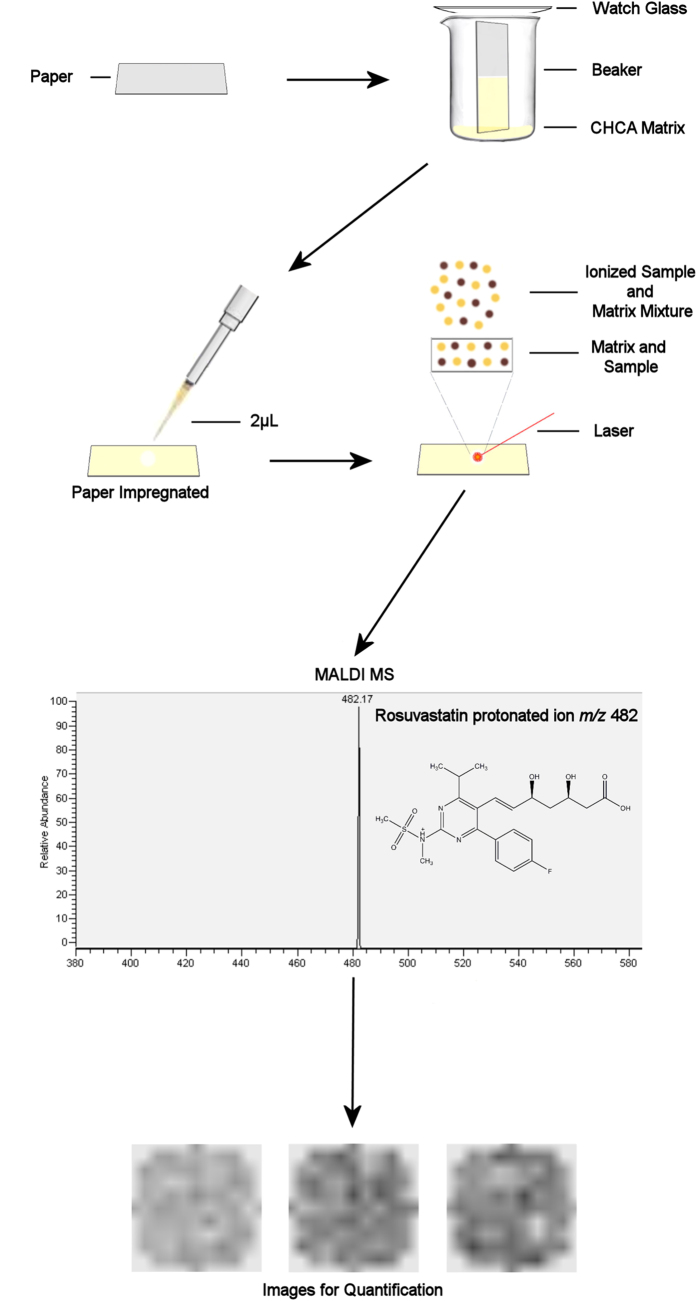
Detailed workflow of the experiments used to carry out MALDI-MSI quantification of rosuvastatin.

A piece of 80 g/m^2^ quantitative filter paper (nominal ash content: 90 μg) with 6-μm pore size (Nalgon, Brazil) was cut into 2.5 × 7.5 cm strips and previously tested to check if any interferences would appear on the selective ion region (data not shown). A solution of α-cyano-4-hydroxycinnamic acid (CHCA) MALDI matrix was prepared in methanol/acetonitrile (50:50) to a final concentration of 25 mg/mL. The filter paper strips were introduced in a glass chamber containing the matrix solution at the bottom and were left to elute completely. Samples from twenty (20) macerated commercial 10-mg generic rosuvastatin tablets of a single manufacturer were diluted in methanol/acetonitrile (50:50) and used for measuring rosuvastatin content. Rosuvastatin standard (Teva API India Limited, India) was diluted in the same solvent system, in different concentrations (0.12, 0.24, 0.36, 0.48 and 0.6 mg.mL^−1^, i.e. 25. 50, 75. 100 and 125%, respectively, as described in the previous subsection above) to build a calibration curve. 2 μL of the final sample solutions were spotted onto the filter paper strips, in triplicates, and sent for mass spectrometry analysis after solvent drying. A brief scheme of the workflow used in the experiments may be found in Fig. 1.

### Mass spectrometry

Experiments were conducted in an LTQ-XL MALDI instrument (Thermo Scientific, San Jose CA) equipped with imaging feature. Typical operating conditions were set as follows: 10 mJ of laser power, 60 μm of raster step size, sample size of 600 × 600 μm, three laser shots per step and collision induced dissociation energy set at 40 units, using helium as the buffer gas for MS/MS measurements. Survey scan analyses were performed at the *m/z* range of 380–600, in the positive ion mode.

### Data workup

Spectral and MS/MS results were analyzed using Mass Frontier software (v. 6.0, Thermo Scientific, San Jose, CA). MSI data were treated using ImageQuest software (Thermo Scientific, San Jose, CA); quantification using MSI was carried out according to de Oliveira *et al.*^20^, where the area (pixels) of the obtained chemical images in grayscale were analyzed using the ImageJ software (National Institutes of Health – Open Source). This software provided arbitrary values regarding pixel intensity: a lighter color in the scale meant less amount of the substance, while a darker color meant a higher amount, much like an HPLC approach, where larger peaks signify larger amounts of the analyte. No data normalization was performed. The obtained arbitrary values provided by ImageJ were then used to plot the analytical curve.

### HPLC

Chromatographic analyses used for comparison in method validation were carried out in a LaChrom Elite, Merck Hitachi HPLC instrument equipped with a Purospher^®^ STAR RP18e 150 × 4.6 mm 5-μm column. Mobile phase was comprised of a 0.05 M formic acid solution and acetonitrile (65:35). Other parameters were as follows: flow rate set at 2.0 mL.min^−1^; injection volume of 10 μL; oven temperature of 35 °C and DAD detector set at λ = 250 nm. Samples were diluted according to the method described above, with a further filtration step through a 0.45-μm PVDF filter. Rosuvastatin retention time was observed at 8.3 min. All validation calculations were performed using the software Validation Manager (version 3.40O, VWR International, Germany).

## Results and Discussion

**Figure 2:**
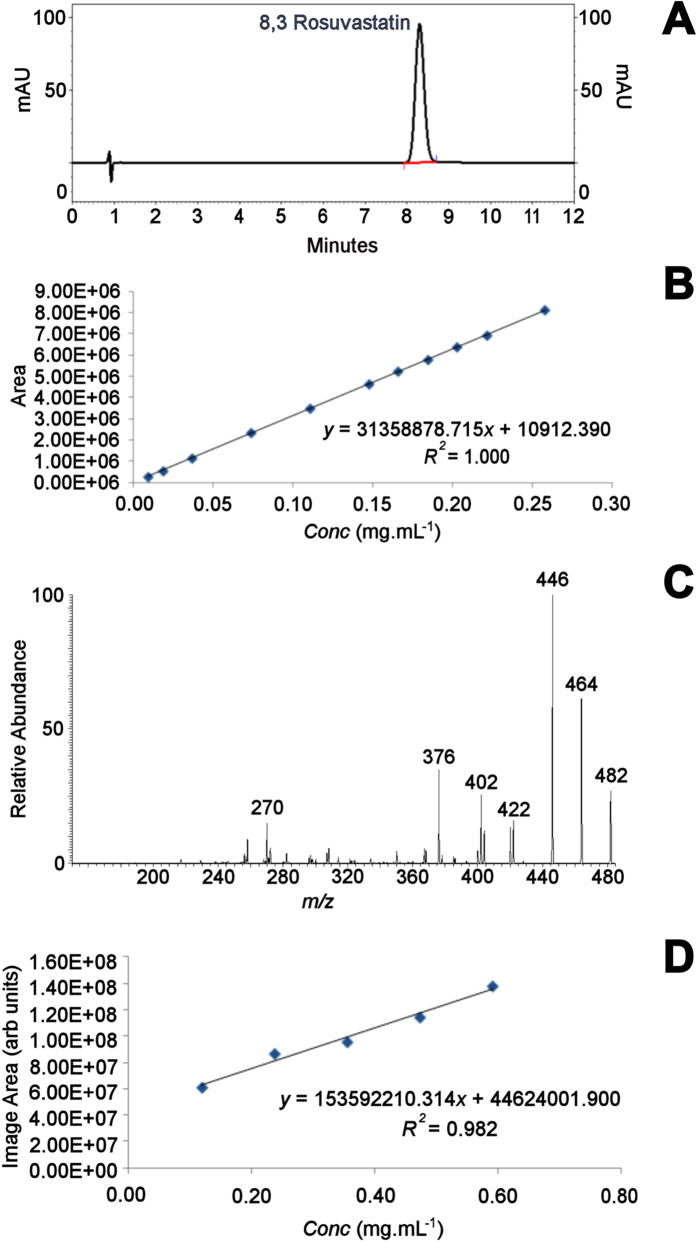
(**A**) Typical chromatogram; (**B**) linearity profile of HPLC; (**C**) typical MS/MS of rosuvastatin and (**D**) linearity profile for MALDI-MSI.

Two main validation parameters were tested to check the power and feasibility of this application: selectivity and linearity. The first was verified through characterization by tandem mass spectrometry (MS/MS; high-resolution figure with structure assignments can be found in supporting information). Figure 1 presents the general workflow of the analytical approach, while Fig. 2 portrays the fragmentation profile of rosuvastatin, with structures assigned to each product ion found, as well as the comparison between linearity curves of MSI and a validated HPLC methodology used for routine quality control.

Linearity check was performed through the establishment of a calibration curve using MSI. After filter paper preparation, extracted 10-mg rosuvastatin tablets obtained from a local manufacturer were used to build a 5-point curve, with concentrations that ranged from 0.12 to 0.6 mg.mL^−1^. Rosuvastatin content was measured using the calibration curve; Table 1 organizes results from the calibration curve, along with the relative standard deviation (RSD, %); and Table 2 observed content for the commercial rosuvastatin tablet. Since RSD values for content were found under the recommended threshold of 2% 21, the MSI strategy may be considered equivalent to HPLC regarding the linearity parameter (HPLC content value for a validated method: 99.8%, RSD 0.804%, data not shown). Estimates of limit of detection (LoD) and limit of quantification (LoQ) were calculated using Validation Manager Software (version 3.40O, VWR International, Germany), and compared to those obtained with HPLC, as seen in Table 1. The calibration curve presented a calculated confidence level of 97.50; values of LoQ were at 0.252 mg.mL^−1^ and LoD <0.01 mg.mL^−1^, calculated according to Huber^21^. The observed recovery in MALDI-MSI for rosuvastatin extraction at 100% (0.47 mg.mL^−1^) is 0.465 mg.mL^−1^ (98.23%). Hence, the obtained results for linearity were deemed validated according to the specifications, as they were within the range for quality control of the finished product (content must be within the range of 90–110%; HPLC: Recovery = 99.8%; LoQ = 4.47 μg.mL^−1^ and LoD = 1.47 μg.mL^−1^, data not shown).

**Figure 3:**
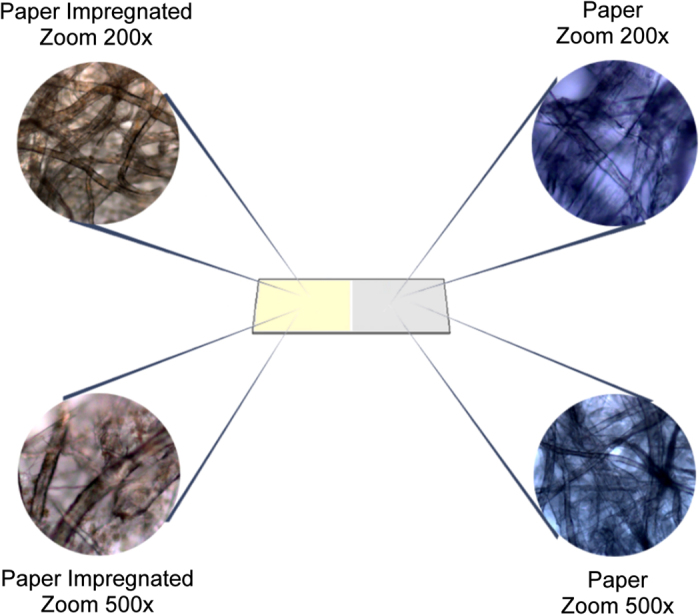
Microscopy images evidencing the paper impregnation by matrix, compared with regular paper.

Using matrix-impregnated filter paper as a support for ionization in MALDI-MSI presented sufficient evidence to be considered an effective method to perform simple and effective quantitative assessment of APIs. The strategy of eluting the paper with a matrix solution prior to sample incorporation eliminates potential problems with homogeneity, which is a considerable drawback in MALDI analyses. Figure 3 illustrates the comparison between a regular filter paper and after impregnation with matrix. It is possible to notice the homogenous dispersion of matrix across the paper fibers, which enhances the ionization potential of molecules deposited onto the paper slide. Several advantages may be therefore inferred from this approach; the use of filter paper, an inexpensive laboratory item, has a standardized grammage and pore size, as well as a very small and controlled amount of impurities (denominated as “ash content”), which are very desirable characteristics for an analytical support. Furthermore, using elution to saturate the paper with matrix is a cost-effective strategy, using very little amounts of solvent and rendering a very homogenous base for ionization, which is evidenced by the linearity observed in the experiments, in addition to an environmental-friendly approach, with little solvent residues generated in the process. Moreover, although HPLC is accurate and precise enough, MALDI-MSI offers a wide range of features to assist in characterization and control of organic molecules that are suitable for API control; MS/MS (tandem MS) emerges as a better and more reliable alternative to ensure method specificity, enhancing the method selectivity. The major limitation of the method, in comparison with HPLC, is in terms of precision and accuracy. This may be explained by the characteristics of the ionization, intrinsic to the method; MALDI uses a pulsed laser to ionize molecules, and the brief interruption in ion formation and, consequently, detection, may directly influence in quantitative results, causing undesired fluctuations and, sometimes, higher RSD values when compared to a conventional approach. However, our experiments evidenced that this phenomenon has very little influence over the results, providing values that are within the acceptable ranges across all analyzed concentrations, in a very straightforward and timely fashion.

## Additional Information

**How to cite this article**: de Menezes, M. *et al.* Capillary-induced Homogenization of Matrix in Paper: A Powerful Approach for the Quantification of Active Pharmaceutical Ingredients Using Mass Spectrometry Imaging. *Sci. Rep.* **6**, 29970; doi: 10.1038/srep29970 (2016).

## Supplementary Material

Supplementary Information

## Figures and Tables

**Table 1:** MALDI-MSI calibration curve data.

Sample volume (mL)	Final volume (mL)	Concentration (mg.mL^−1^)	Area (arb units)	RSD (%)
0.25	10.00	0.11832	61282934	5.999
0.50	10.00	0.23665	86460116	1.470
0.75	10.00	0.35497	95403564	1.176
1.00	10.00	0.47329	114391013	2.573
1.25	10.00	0.59162	138185037	1.307

**Table 2:** Observed values for content on the commercial rosuvastatin tablet.

Sample dilution factor	Mean weight (tablets, mg)	Concentration (mg.mL^−1^)	Area (arb. units)	Actual mass (mg)	Content (%)	RSD (%)
20	102.60	0.465	116028109	9.298	98.23	1.950
