# Supplementary material for: Capillary-induced Homogenization of Matrix in Paper: A Powerful Approach for the Quantification of Active Pharmaceutical Ingredients Using Mass Spectrometry Imaging
Source: Sci Rep. 2016 Jul 21;6:29970. doi: 10.1038/srep29970 (PMC4954946; doi:10.1038/srep29970)
Supplement: Supplementary Information [file srep29970-s1.pdf]

# Capillary-induced Homogenization of Matrix in Paper: A Powerful Approach for the Quantification of Active Pharmaceutical Ingredients Using Mass Spectrometry Imaging

*Maico de Menezes, Diogo Noin de Oliveira and Rodrigo Ramos Catharino**

## Supporting information

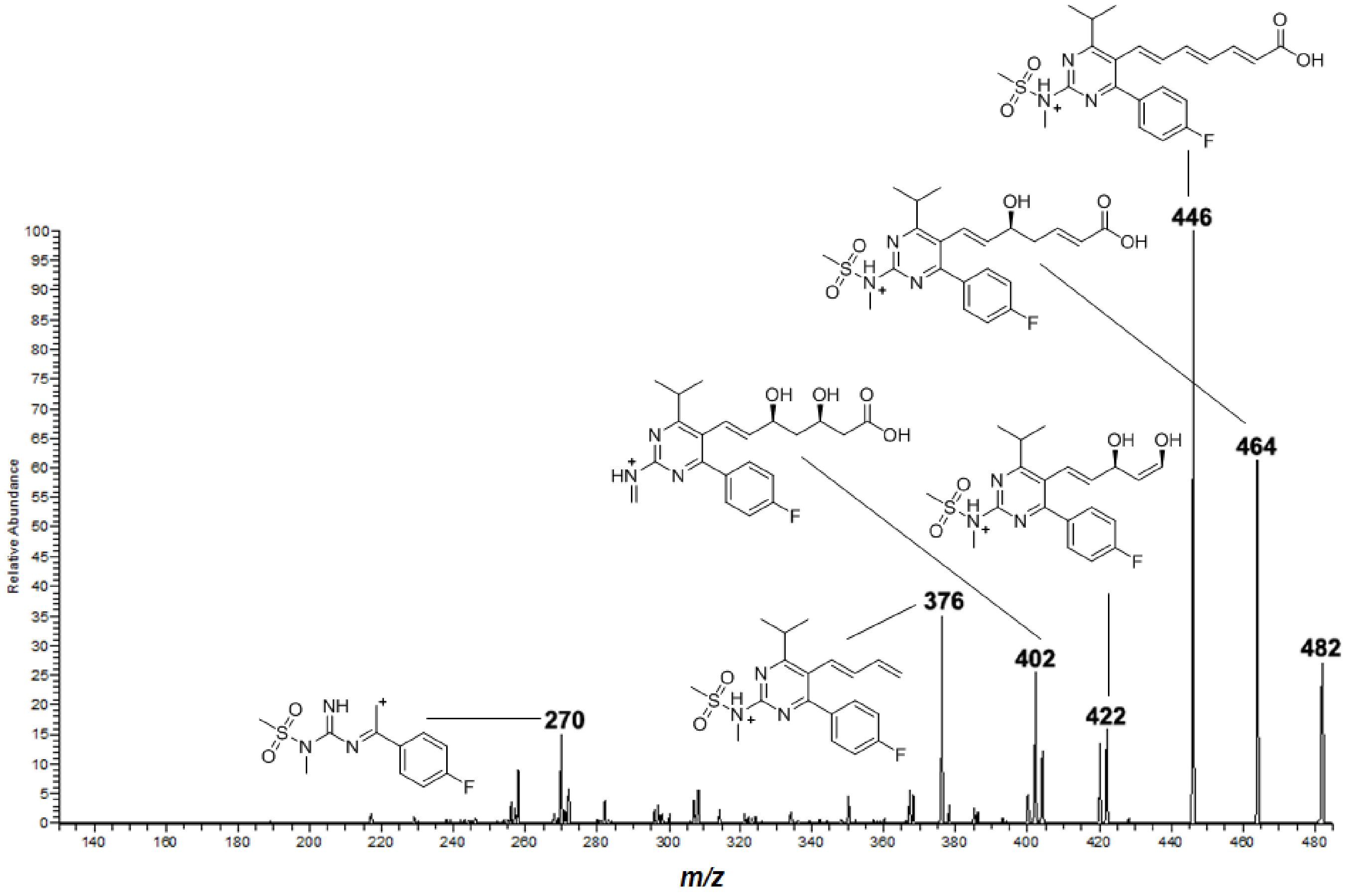


**Figure S1.** MS/MS spectrum of Rosuvastatin (ion at *m/z* 482 $[M+H]^+$). All main product ions are identified to characterize the active pharmaceutical ingredient.
